# Flourishing and its influencing factors among maintenance hemodialysis patients in Shanghai, China: a cross-sectional study

**DOI:** 10.3389/fpsyt.2025.1480308

**Published:** 2025-03-27

**Authors:** Xiao Qing Zeng, Hong Li Yan, Yue Qin Qian, Yong Qi Li, Jie Yang, Yan Lin Gong, Yi Liu, Li Jing Chen, Jing Wu, Jing Chu

**Affiliations:** ^1^ Faculty of Nursing, Naval Medical University, Shanghai, China; ^2^ School of Health Management, Southern Medical University, Guangzhou, Guangdong, China; ^3^ Ruijin Hospital, Shanghai Jiao Tong University School of Medicine, Shanghai, China

**Keywords:** maintenance hemodialysis, chronic kidney disease, flourishing, PERMA, social support, quality of life

## Abstract

**Background:**

Maintenance hemodialysis (MHD) patients face substantial psychological challenges that impacting their overall quality of life. Flourishing, a concept within positive psychology, refers to a state of mental well-being and personal growth. Despite its importance, the factors influencing flourishing in MHD patients remain underexplored.

**Aim:**

This cross-sectional study aimed to assess flourishing levels among MHD patients in Shanghai, China, and identify sociodemographic, disease-related, and psychological factors associated with flourishing, with implications for targeted interventions.

**Method:**

From October to November 2022, 376 MHD patients across four hospitals completed validated scales measuring flourishing (PERMA Profiler), personality traits (TIPI-C), regulatory emotional self-efficacy (RES), perceived social support (PSSS), and quality of life (EQ-5D). Statistical analyses, including regression analysis, were used to identify factors associated with flourishing.

**Results:**

The mean flourishing score was 6.28 ± 1.763, indicating moderate levels compared to general populations. Full-time employment (β = 0.749, p = 0.033), retirement (β = 0.675, p = 0.043), social support from friends/others (β = 0.039, p < 0.001), conscientiousness (β = 0.133, p < 0.001), and better quality of life (β = 1.281, p = 0.001) emerged as significant positive predictors. Conversely, longer dialysis duration (ρ = -0.135, p = 0.009) and higher perceived disease impact (β = -0.084, p = 0.268) were negatively associated with flourishing.

**Conclusions:**

The findings highlight the complex interplay between sociodemographic, disease-related, and psychological factors in influencing the flourishing of MHD patients. The level of flourishing in MHD patients’ needs to be improved. Developing targeted interventions based on these relevant factors improves quality of life and thus contributes significantly to the well-being of MHD patients.

## 1 Introduction

Chronic kidney disease (CKD) is a global public health crisis, with over 553,000 patients in China relying on maintenance hemodialysis (MHD) for survival (1). While MHD sustains life, its physical, psychological, and socioeconomic burdens—including thrice-weekly treatments, dietary restrictions, employment disruptions, and financial strain—profoundly diminish patients’ quality of life (QoL) (2, 3). Consequently, MHD patients exhibit elevated rates of anxiety, depression, and social isolation (2, 4–6), underscoring the urgent need to shift from mere survival to holistic well-being.

Flourishing, defined as a state of optimal mental health characterized by positive emotions, purposeful engagement, and fulfilling relationships (7), has become as a critical target in chronic disease management. Grounded in Seligman’s PERMA framework (Positive emotion, Engagement, Relationships, Meaning, Accomplishment) (8), flourishing transcends the absence of pathology by emphasizing resilience and growth. Recent work by VanderWeele (9, 10) and House et al. (11) highlights its relevance in healthcare, particularly for populations navigating lifelong treatments like MHD. For these patients, flourishing may mitigate treatment-related distress and enhance adaptive coping (12). However, despite its theoretical promise, empirical data on flourishing in MHD patients remain scarce.

Personality traits, which are stable psychological characteristics, influence all aspects of patient behavior and are challenging to alter (13, 14). The Big Five Theory of Personality categorizes human personality traits into five dimensions: Neuroticism, Extraversion, Openness to Experience, Agreeableness, and Conscientiousness (15). Previous studies have shown that flourishing is correlated with personality traits. Flourishing is significantly positively correlated with Conscientiousness, Agreeableness, Extraversion, and Openness, while it is significantly negatively correlated with Neuroticism (16). Understanding the personality traits of MHD patients may provide a foundation for personalized interventions aimed at enhancing their well-being.

Regulatory emotional self-efficacy (RES), defined as an individual’s confidence in managing emotional states, and consists perceived self-efficacy in managing anger/irritation (ANG), despondency/distress (DES), and positive affect (POS) (17). Under the dual pressure of physical and psychological stress imposed by their condition, MHD patients are prone to negative emotions such as anxiety and fear (18). RES plays a crucial role in shaping negative emotions (anger, depression and anxiety) and prosocial aggressive behavior (19). Related studies have shown that regulatory emotional self-efficacy is conducive to individuals experiencing more positive emotions, and individuals are prone to positive social interaction experiences (20). However, the relationship between RES and flourishing remains unclear and needs to be further explored.

Social support refers to the emotional and material assistance that an individual receives from social relationships, including family members, friends, significant others, and various organizations (21). MHD patients need to receive lifelong treatment, and social support is indispensable. Given the extensive impact of CKD on all aspects of life, social support from family, friends, and other significant individuals can help patients better adapt to their condition and self-management (22). Investigating the effects of different types of social support on flourishing can provide valuable insights into the needs of MHD patients and inform the development of targeted interventions.

As a relatively new and important construct in positive psychology, flourishing merits significant attention. The study aimed to assess flourishing levels among MHD patients in Shanghai, China, and identify sociodemographic, disease-related, and psychological factors associated with flourishing, with implications for targeted interventions. The present study proposed the following hypotheses:

H1: Sociodemographic factors (education, employment, marital status) are positively associated with flourishing.
H2: Disease-related factors (dialysis duration, perceived disease impact, quality of life) are associated with flourishing.
H3: Psychological factors (regulatory emotional self-efficacy, social support, personality traits) are positively associated with flourishing, except for neuroticism, which is negatively associated.

## 2 Methods

### 2.1 Procedure and ethics

This study was designed as a cross-sectional, multi-center investigation. Data collection occurred between October 1st and November 30th, 2022, targeting maintenance hemodialysis (MHD) patients from four hospitals in Shanghai. Participants were invited to complete a paper-based questionnaire, either independently or with the assistance from researchers during dialysis sessions. The study adhered to all relevant guidelines, regulations, and the principles of Declaration of Helsinki. Ethical approval was granted by the Committee on Ethics of Medicine, Naval Medical University. Informed consent was obtained from each participant, and when necessary, from their legal guardian. To ensure patient privacy and anonymity, no identifying information, including photographs, was collected. Participation was voluntary, with no monetary or material incentives provided.

### 2.2 Participants and sample

A convenience sampling method was employed to recruit participants, with specific eligibility criteria established. Inclusion criteria included individuals diagnosed with End-Stage Renal Disease (ESRD) according to the International Classification of Diseases (ICD-10), aged 18 years or older, who had been undergoing hemodialysis for a minimum of three months, possessed no perception disorders or communication problems, and were willing to provide informed consent. Exclusion criteria encompassed individuals who had undergone major surgery, suffered from severe psychiatric disorders (Schizophrenia, Bipolar Disorder, Schizoaffective Disorder, Persistent Delusional Disorder, Mental Disorders due to Epilepsy, Mental Retardation with Associated Mental Disorders), severe heart, liver, or respiratory failure, or malignant tumors. A power analysis using G*Power 3.1 was conducted to determine the target sample size required to detect effects in the planned analyses. For multiple linear regression with 19 predictor variables, an alpha of 0.05, and a power of 0.80, the recommended sample size was 133 participants. The obtained sample of 376 individuals exceeded this threshold, ensuring sufficient statistical power.

### 2.3 Measures

#### 2.3.1 Sociodemographic characteristics questionnaire

The demographic questionnaire that was developed by the researchers contained questions on sociodemographic and disease-related characteristics of the patients such as gender, age, education, marital status, primary caregiver, employment, dialysis duration, presence of a co-morbid disease, kidney transplant experience, self-perceived degree of knowledge of disease-related information, self-perceived burden of medical expenses for disease, self-perceived degree of impact of disease on life.

#### 2.3.2 PERMA profiler of Chinese version

The PERMA scale is a 23-item measure of flourishing developed by Kern et al. (8). Widely utilized in the United States, Australia, Korea, Japan, and other global populations, it has been adapted by our research group into a Chinese version, demonstrating commendable reliability (8, 23–26). The scale encompasses 15 items measure PERMA (Positive emotions, Engagement, Relationships, Meaning and Accomplishments). There are additional three item subscales examine Overall Happiness, Loneliness, Physical Health and Negative Emotion. Each item is rated on a Likert-type scale ranging from 0 to 10. Dimension scores were calculated as the average score of the corresponding items. The flourishing score is derived from adding the PERMA Total to the Overall Happiness item. The research team translated the PERMA scale into Chinese and conducted an exploratory factor analysis on the survey results from 376 samples. The Chinese version retained all original items and factor analysis identified two dimensions: Positive experience and Self-accomplishment. The original subdomains of Positive emotions and Relationships were consolidated into a unified “positive experience” dimension, while Engagement, Meaning, and Accomplishment were combined to form a “Self-accomplishment” dimension. The two dimensions obtained by EFA were used for model fitting, and the results of model fitting indices showed χ2/df=3.234, RMSEA=0.095, GFI=0.858, CFI=0.894, IFI=0.895, and TLI=0.875. The Cronbach’s α of the scale in this study was 0.895 (95% CI [0.879, 0.910]).

#### 2.3.3 Ten-item personality inventory in China

The Ten-Item Personality Inventory in China (TIPI-C), developed by Gosling et al. (27), serves as a tool to assess Big-Five personality traits. Jinde Li translated the TIPI into the Chinese version (28). Comprising 10 items distributed across five dimensions (E-Extroversion, A-Agreeableness, C-Conscientiousness, ES-Emotional Stability and O-Openness), the TIPI utilizes two items for each Big-Five personality dimension. Notably, items 2, 4, 6, 8, and 10 should be reverse-coded when computing the dimension score. Responses were recorded on a Likert scale ranging from 1 to 7 (1=strongly disagree, 7=strongly agree), with higher scores indicating a greater expression of the respective trait. The Cronbach’s α of the scale in this study was 0.6439 (95%CI [0.608, 0.678]). CFA proved that the goodness-of-fit indicators were acceptable (28).

#### 2.3.4 Regulatory emotional self-efficacy scale

The Regulatory Emotional Self-Efficacy Scale (RES), initially designed by Caprara et al. and later revised by Yu Guoliang et al. (29), comprises a total of 12 questions distributed across three dimensions: Perceived self-efficacy in expressing positive affect (POS, 4 items), Perceived self-efficacy in managing despondency (DES, 5 items), and Perceived self-efficacy in managing anger (ANG, 3 items). Respondents provide ratings for each item on a 5-point Likert scale, ranging from 1 to 5. A higher score indicates a higher level of regulatory emotional self-efficacy exhibited by the investigator. The Cronbach’s α of the scale in this study was 0.872 (95% CI [0.850, 0.894]). Goodness-of-fit indicators were within acceptable levels (29).

#### 2.3.5 Perceived social support scale

The Perceived Social Support Scale (PSSS) was employed to assess an individual’s perceived social support (30). Each participant’s responses were evaluated using a 12-item scale, comprising two subscales: family support (4 items) and friends and important others support (8 items). Participants provided responses to each item on a 7-point Likert scale. The scores of items within each dimension were aggregated, with higher scores indicating a greater perception of social support. The Cronbach’s α of the scale in this study was 0.875 (95%CI [0.853, 0.897]). Fit measures fell within the range of acceptability (30).

#### 2.3.6 EuroQol five-dimensional questionnaire

The EuroQol five-dimensional questionnaire (EQ-5D), a globally recognized patient-reported outcome instrument renowned for its succinct and lucid items, is widely employed for health measurement and valuation due to its effective assessment capabilities. It has also become a commonplace tool for evaluating the quality of life among patients grappling with chronic kidney disease (31). The EQ-5D comprises two parts: a multidimensional health classification system and a visual analog assessment system. For this study, only the Multidimensional Health Classification System was utilized for assessment, considering the potential impact of personal researcher perception on the scoring values. The EQ-5D evaluates health across five dimensions—mobility (MOB), self-care (SC), usual activities (UA), pain/discomfort (PA), and anxiety/depression (MOOD)—with three severity levels in each dimension. It has established a corresponding utility value point system in China (1=no problems, 2=some/moderate problems, 3=extreme problems/unable) (32). The Cronbach’s α of the scale in this study was 0.766 (95%CI [0.735, 0.797]).

### 2.4 Statistical analysis

All statistical analyses were conducted using IBM SPSS 27.0. Data were initially entered into EpiData 3.0 to ensure accuracy and minimize entry errors, after which the dataset was imported into SPSS for further analysis. Descriptive statistics, including frequency distributions, means, and standard deviations (SD), were used to summarize participants’ sociodemographic and clinical characteristics.

Univariate analyses were performed to assess associations between flourishing scores and independent variables. For dichotomous variables, independent samples t-tests were applied when normality (assessed by the Shapiro-Wilk test) and homogeneity of variance (verified by Levene’s test) assumptions were satisfied; otherwise, the non-parametric Mann-Whitney U test was used. For multinomial categorical variables, one-way ANOVA was utilized when data met normality and equal variance requirements, while the Kruskal-Wallis H test was adopted as the non-parametric alternative when these assumptions were violated. Pearson correlation coefficients were calculated to assess linear relationships between continuous variables with normal distribution and homoscedasticity, whereas Spearman’s rank correlation coefficients were computed for variables violating these parametric assumptions.

A stratified stepwise regression model was constructed. Independent variables were grouped into three blocks: sociodemographic variables (e.g., education, marital status, employment), disease-related variables (e.g., dialysis duration, comorbidities, perceived disease impact), and psychological variables (e.g., personality traits, regulatory emotional self-efficacy, social support). Blocks 1 and 2 were entered using the enter method, while Block 3 was analyzed using stepwise selection (entry criteria: p < 0.05; removal criteria: p > 0.10). Effect sizes were calculated using Cohen’s f² for regression models, partial η² for ANOVA, and Cohen’s d or r for t-tests and correlations (33). The significance level was set at α = 0.05 (two-tailed).

## 3 Results

### 3.1 Participant’ s sociodemographic and disease-related characteristics

A total of 406 questionnaires were collected, of which 376 were deemed valid, resulting in a questionnaire validity rate of 92.61%. Among the 376 participants, the mean age was 58 ± 13.403 years, with an age range from 22 to 97 years. The mean duration of dialysis was 7 ± 6.601 years, with a range from 4 months to 34 years. Thirty of them (8.0%) had undergone renal transplantation. The number of patients surveyed with chronic diseases other than chronic kidney disease was 80.6% (203). The rest of the information is shown in **Table 1**.

**Table 1:** Descriptive statistics for sociodemographic and clinical characteristics of participants (n=376).

Characteristics	*N *(%)	Category	*N *(%)
Gender		Number of co-morbid disease	
Male	220 (58.5)	None	73 (19.4)
Female	156 (41.5)	1	141 (37.5)
Education		2	99 (26.3)
Elementary or less	11 (2.9)	3	41 (10.9)
Middle graduate	100 (26.6)	≥4	22 (5.9)
High graduate	130 (34.6)	Self-perceived degree of knowledge of disease-related information	
College or higher	135 (35.9)	Very much	28 (7.4)
Marital status		Much	123 (32.7)
Married	300 (79.8)	Somehow	219 (58.2)
Unmarried	40 (10.6)	Not at all	6 (1.6)
Widowed	17 (4.5)	Self-perceived burden of medical expenses for disease	
Divorced	19 (5.1)	Very light	48 (12.8)
Primary caregiver		Mild	100 (26.6)
Parent/Child	66 (17.6)	Moderate	158 (42.0)
Spouse	150 (39.9)	Severe	55 (14.6)
Self	151 (40.2)	Very severe	15 (4.0)
Other	9 (2.4)	Self-perceived degree of impact of disease on life	
Employment		Very light	11 (2.9)
Full-time job	77 (20.5)	Mild	56 (14.9)
part-time job	14 (3.7)	Moderate	171 (45.5)
Retirement	241 (64.1)	Severe	109 (29.0)
Unemployment	44 (11.7)	Very severe	29 (7.7)

### 3.2 Flourishing levels in maintenance hemodialysis patients

The mean flourishing score was 6.28 ± 1.763 (scale range: 0–10), indicating moderate levels compared to general population (typically >7.0) (8). The result suggest there remains substantial potential for enhancing mental health outcomes within this surveyed population. Among PERMA dimensions, positive experience scored highest (6.74 ± 1.727), while self-accomplishment was the lowest (5.88 ± 2.135). Negative emotion and loneliness scores were 3.65 ± 2.354 and 3.84 ± 3.089, respectively, reflecting significant psychological distress. **Table 2** summarizes PERMA-Profiler scores.

**Table 2:** PERMA-profiler scores of MHD patients.

Dimension	M (P25, P75)	$\bar{X}$ ± *SD*
Positive experience	6.83 (5.67, 8.00)	6.74 ± 1.727
Self-accomplishment	6.11 (4.47, 7.67)	5.88 ± 2.135
Negative emotion	3.50 (1.67, 5.33)	3.65 ± 2.354
Physical health	5.67 (4.33, 7.00)	5.57 ± 2.006
Loneliness	3.00 (1.00, 6.00)	3.84 ± 3.089
Overall happiness	8.00 (6.00, 9.00)	7.10 ± 2.212
Flourishing	6.38 (5.19, 7.73)	6.28 ± 1.763

The score of flourishing was calculated as an average of Positive emotion, Accomplishment and Overall happiness.

### 3.3 Sociodemographic and disease-related characteristics associated with flourishing

Flourishing scores varied significantly across sociodemographic and disease-related factors. The level of flourishing exhibited variations based on factors such as education, marital status, employment, dialysis duration, the presence of co-morbid diseases, the degree of knowledge regarding disease-related information, the financial burden of medical expenses, and the impact of the disease on life. Notably, dialysis duration demonstrated a significant negative correlation with the level of flourishing (ρ=-0.135, P=0.009) **Table 3** presents the descriptive information for participant’s sociodemographic and disease-related characteristics by score of flourishing.

**Table 3:** Descriptive information for participant’s sociodemographic and disease-related characteristics by score of flourishing.

Variables	Score of Flourishing		Statistic	*P* value	Effect Size	*Post Hoc* Multiple Comparisons
	M (P25, P75)	$\bar{X}$ ± *SD*				
Gender			-1.832^1^	0.678	0.192	
Male	6.31 (4.95, 7.63)	6.14 ± 1.832				
Female	6.43 (5.50, 7.75)	6.48 ± 1.646				
Education			6.254^2^	<0.001	0.048	College degree or higher>Middle school graduate; Elementary school degree or lower
Elementary or less	4.88 (3.81, 7.50)	5.11 ± 2.410				
Middle graduate	5.84 (4.56, 7.43)	5.86 ± 1.770				
High graduate	6.31 (5.31, 7.75)	6.27 ± 1.685				
College or higher	6.81 (5.75, 7.94)	6.69 ± 1.673				
Marital status			8.444^3^	0.038	0.023	Married>Widowed
Married	6.44 (5.27, 7.75)	6.37 ± 1.742				
Unmarried	6.28 (5.03, 7.36)	6.03 ± 1.909				
Widowed	5.13 (4.13, 5.91)	5.15 ± 2.069				
Divorced	6.38 (5.56, 7.19)	6.30 ± 1.078				
Primary caregiver			4.588^3^	0.205	0.012	
Parent/Child	6.34 (4.86, 7.11)	6.00 ± 1.619				
Spouse	6.25 (5.05, 7.70)	6.23 ± 1.745				
Self	6.63 (5.38, 7.75)	6.48 ± 1.771				
Other	4.69 (4.06, 8.84)	5.68 ± 2.643				
Employment			14.883^3^	0.002	0.040	Full-time job > Retirement, Part-time job, Unemployment
Full-time job	6.94 (5.94, 7.81)	6.86 ± 1.437				
Part-time job	5.63 (4.02, 6.52)	5.29 ± 1.666				
Retirement	6.38 (4.97, 7.75)	6.23 ± 1.785				
Unemployment	5.94 (4.73, 7.31)	5.81 ± 1.914				
Number of co-morbid			7.622^2^	<0.001	0.020	None> (≥3);1> (≥2);
None	6.69 (5.66, 7.88)	6.69 ± 1.631				
1	6.75 (5.63, 7.94)	6.67 ± 1.582				
2	5.94 (5.13, 7.38)	5.95 ± 1.807				
3	5.19 (4.13, 6.88)	5.49 ± 1.879				
≥4	5.53 (4.02, 6.59)	5.36 ± 1.877				
Kidney transplant			0.039^4^	0.969	0.074	
Yes	5.94 (5.00, 8.33)	6.28 ± 2.121				
No	6.38 (5.19, 7.69)	6.28 ± 1.732				
Self-perceived degree of knowledge of disease-related information			5.259^2^	0.001	0.041	Very much>Somehow, Not at all
Very much	7.50 (5.89, 8.47)	7.20 ± 1.564				
Much	6.50 (5.38, 7.81)	6.45 ± 1.715				
Somehow	6.19 (4.94, 7.44)	6.11 ± 1.757				
Not at all	4.16 (3.23, 6.91)	4.77 ± 2.007				
Self-perceived burden of medical expenses for disease			18.355^3^	0.001	0.012	Very light, Mild>Severe
Very light	6.78 (5.94, 8.30)	6.94 ± 1.787				
Mild	6.63 (5.53, 7.94)	6.59 ± 1.598				
Moderate	6.31 (4.94, 7.59)	6.18 ± 1.665				
Severe	5.69 (4.38, 7.13)	5.59 ± 1.900				
Very severe	5.75 (4.50, 7.69)	5.63 ± 2.208				
self-perceived degree of impact of disease on life			5.578^2^	<0.001	0.057	Mild>Very severe; Mild, Moderate>Severe
Very light	6.13 (5.69, 8.81)	6.86 ± 1.828				
Mild	6.97 (5.94, 7.98)	6.90 ± 1.402				
Moderate	6.63 (5.38, 7.75)	6.45 ± 1.679				
Severe	6.00 (4.50, 7.22)	5.78 ± 1.847				
Very severe	5.75 (4.75, 7.19)	5.73 ± 1.984				
Age	6.38 (5.19, 7.73)	58.00 ± 13.403	-0.060^5^	0.246	-0.060	
Dialysis duration	6.38 (5.19, 7.73)	7.00 ± 6.601	-0.135^5^	0.009	-0.135	

^1^ Independent Samples t-test presented; ^2^ANOVA presented; ^3^ Kruskal-Wallis H test presented; ^4^ Mann-Whitney U test presented; ^5^ Spearman rank coefficient; M, Median; P25/P75, 25th/75th percentiles; SD, Standard Deviation.

### 3.4 Correlation analysis of flourishing and personality traits, regulatory emotional self-efficacy, social support and quality of life

**Figure 1:**
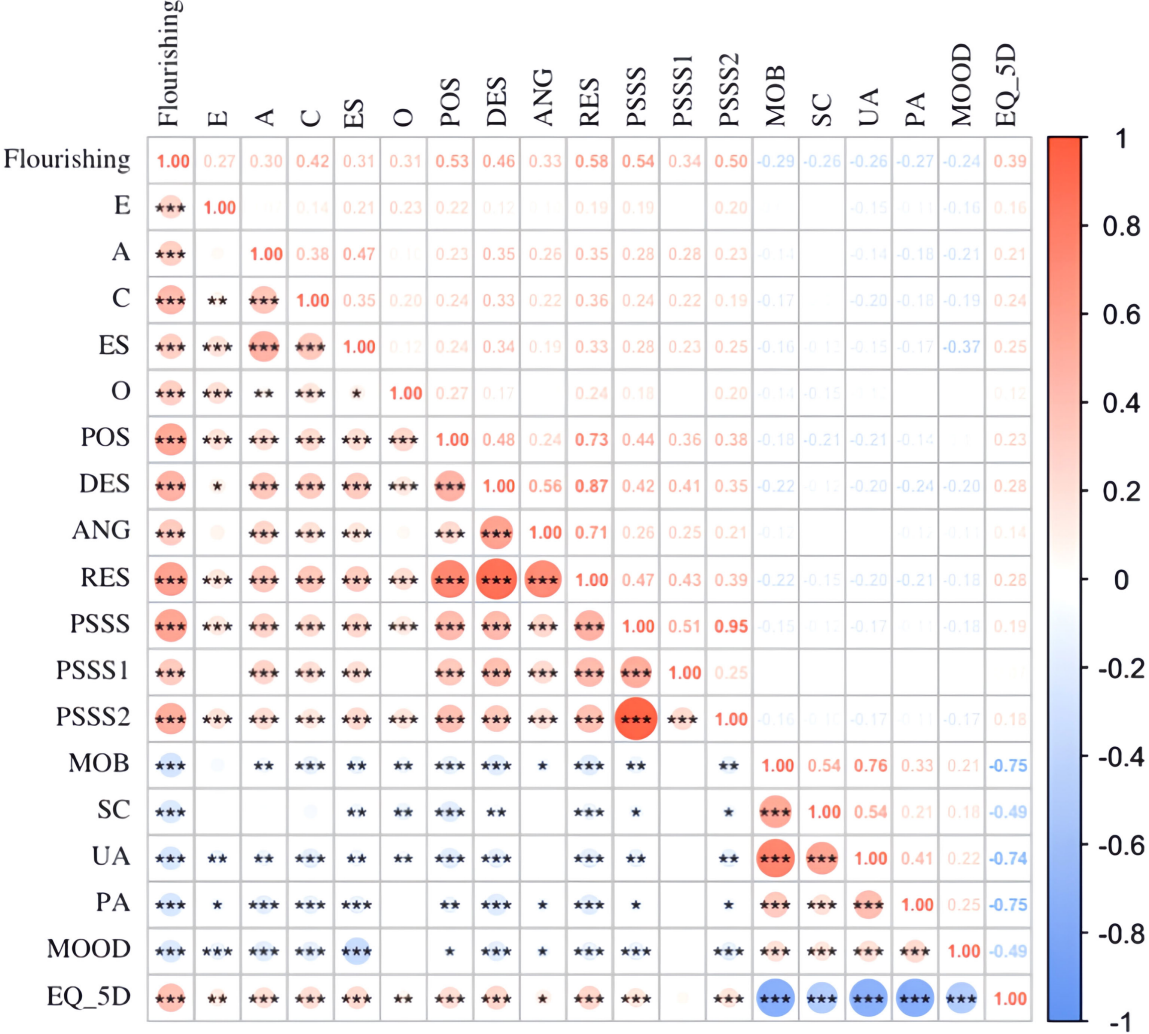
Bivariate correlations of study variable (n=376). E represents extroversion; A represents agreeableness; C represents conscientiousness; ES represents emotional stability; O represents openness; POS represents Perceived self-efficacy in expressing positive affect; DES represents perceived self-efficacy in managing despondency; ANG represents perceived self-efficacy in managing anger; RES represents regulatory emotional self-efficacy; PSSS represents social support; PSSS1 represents family support; PSSS2 represents friends and important others support; MOB represents mobility; SC represents self-care represents; UA represents usual activities; PA represents pain/discomfort; MOOD represents anxiety/depression; EQ_5D represents quality of life.^*^ *P*<0.05, ^**^ *P*<0.01, ^***^ *P*<0.001.

Spearman’s rank correlation (ρ) was used due to non-normality. Spearman’s correlation analysis revealed significant associations between flourishing levels and psychological variables (see **Figure 1**). All five traits (Extraversion, Agreeableness, Conscientiousness, Emotional Stability, Openness) showed positive correlations (ρ= 0.27–0.42, p < 0.001). RES total score (ρ = 0.58, p < 0.001) and its subdimensions (p < 0.05) were strong positive associated to Flourishing. Both family support (p = 0.038) and support from friends/others (p < 0.001) contributed to flourishing.

### 3.5 Regression analysis of flourishing in maintenance hemodialysis patients

Stratified stepwise regression identified 10 variables explaining 56.7% of the variance in flourishing (adjusted R² = 0.567, F = 25.591, p < 0.001), with effect size of f² ≈ 1.31. The Durbin-Watson statistic of the model was 1.721, with the tolerance ranging from 0.140 to 0.883. The detailed results are outlined in **Table 4**. The variables of Agreeableness (A), Emotional Stability (ES), and Perceived self-efficacy in managing despondency (DES) were ultimately excluded from the final model. The following variables were identified as positive associated with flourishing: employed full-time (β=0.749, *P*<0.05), retirement (β=0.675, *P <*0.05), Perceived self-efficacy in expressing positive affect (β=0.137, *P <*0.001), Friends and others support (β=0.039, *P <*0.001), Conscientiousness (β=0.133, *P <*0.001), EQ-5D (β=1.281, *P* =0.001), Openness (β=0.091, *P* =0.001), Perceived self-efficacy in managing anger (β=0.055, *P <*0.05), Extroversion (β=0.062, *P <*0.05), Family support (β=0.032, *P <*0.05). **Supplementary Table 1** shows the value labels of characteristics.

**Table 4:** Results of stratified regression in each group.

In-model variables	Block 1			Block 2			Block 3		
	*β*	t	*P*	*β*	t	*P*	*β*	t	*P*
Constant	3.099	4.484	<0.001	6.672	7.670	<0.001	-2.469	-2.868	0.004
Education	0.395	3.556	<0.001	0.280	2.569	0.011	0.009	0.107	0.915
Marital status (‘Widowed’ as a reference)									
Married	0.989	2.304	0.022	0.879	2.148	0.032	0.350	1.161	0.246
Unmarried	0.539	1.020	0.308	0.524	1.040	0.299	0.070	0.190	0.850
Divorced	0.721	1.235	0.218	0.775	1.388	0.166	0.289	0.705	0.481
Employment (‘Employed part-time’ as a reference)									
Employed full-time	1.458	2.936	0.004	0.863	1.792	0.074	0.749	2.135	0.033
Retirement	1.122	2.390	0.017	0.772	1.712	0.088	0.675	2.030	0.043
Unemployment	0.671	1.276	0.203	0.460	0.915	0.361	0.539	1.464	0.144
Dialysis duration				-0.031	-2.294	0.022	-0.010	-1.018	0.309
Number of co-morbid disease				-0.262	-3.237	0.001	-0.047	-0.790	0.430
Burden of expenses				-0.138	-1.465	0.144	-0.038	-0.552	0.581
Knowledge of disease information				0.409	3.070	0.002	0.052	0.530	0.597
self-perceived degree of impact of disease on life				-0.234	-2.318	0.021	-0.084	-1.109	0.268
POS							0.136	6.582	<0.001
Friends and others support							0.039	5.839	<0.001
Conscientiousness							0.133	4.477	<0.001
EQ-5D							1.281	3.391	0.001
Openness							0.091	3.245	0.001
ANG							0.055	2.376	0.018
Extroversion							0.062	2.818	0.005
Family support							0.032	2.086	0.038
Adjusted R^2^		0.079			0.170			0.567	
F		5.591			7.387			25.591	
*P*		<0.001			<0.001			<0.001	

## 4 Discussion

### 4.1 Flourishing of MHD patients needs improvement

This study assessed the level of flourishing among MHD patients using the PERMA framework and explored its influencing factors. The mean flourishing score remains significantly lower than general population norms (>7.0) (8). Notably, the “self-accomplishment” dimension scored lowest, likely reflecting the profound disruption of life goals caused by dialysis schedules and physical limitations. These results underscore that merely sustaining survival is insufficient; enabling patients to thrive requires addressing psychological and social barriers.

Seligman defines subjective well-being in five domains. Positive emotions are an important part of well-being. Research indicates that MHD patients are prone to distress from disease symptoms and anxiety about complications, which negatively impacts their emotional well-being. The physical limitations and social role changes imposed by dialysis may exacerbate negative emotions. (34). Engagement, defined as a deep psychological involvement characterized by intense concentration, is also compromised due to the physical weakness experienced by MHD patients (8). This weakness often hinders daily activities, leaving patients fatigued and may be less engaged in life (35). Relationships are fundamental to life and including social ties, social networks, and received support. MHD patients receive dialysis treatments 2-3 times per week (36), which require patients to travel frequently to and from the hospital, affecting the quality of their socialization and the interpersonal relationships of MHD patients. The “meaning” aspect, which involves having a sense of purpose and feeling that one’s life is valuable, is particularly affected in MHD patients due to the lifelong nature of treatment. The numerous restrictions imposed by dialysis, such as dietary limitations, reduced ability to travel, and fatigue at work, could lead to a diminished sense of life’s meaning (37). The “accomplishment” aspect scored lowest, likely reflecting the profound disruption of life goals caused by dialysis schedules and physical limitations (38).

### 4.2 Factors influencing flourishing in MHD patients

#### 4.2.1 Sociodemographic factors

The study found that several sociodemographic factors influence the flourishing among MHD patients, supporting Hypothesis 1 (H1). Higher education levels and a greater understanding of disease-related information were associated with higher levels of flourishing, consistent with previous research findings (39, 40). Patients with higher education tend to have better self-regulation skills, which contributes to improved quality of life and mental health (41). The study’s findings suggest that intimate relationships stemming from marriage, stable employment status positively influence the flourishing of MHD patients. Previous research has indicated that MHD patients with a partner feel more cared for and supported, and a stable and warm intimate relationship promotes their mental health (42). Employment status emerged as the sole sociodemographic variable entering the hierarchical linear regression equation in this study. Univariate analysis results further highlight that MHD patients with full-time employment exhibit the highest level of flourishing. Despite the impact of MHD on their daily lives, individuals with full-time jobs can still engage in work activities, derive a sense of value from completing tasks, and thereby bolster their mental health (43).

#### 4.2.2 Disease-related factors

The duration of MHD treatment, the perceived impact of the disease on life, and the financial burden of medical expenses were all found to be negatively associated with flourishing, supporting Hypothesis 2 (H2). These findings align with existing literature, which suggests that prolonged dialysis leads to weakened physiological functions, decreased immunity, and an increased burden of chronic diseases, all of which negatively impact quality of life and flourishing (44). With the prolonged duration of MHD, patients experience weakened physiological functions, decreased immunity, increased chronic diseases, and a heightened impact on their daily lives (45). Consequently, the quality of life is further compromised, adversely affecting the level of flourishing. Existing studies corroborate the significant impact of patients’ quality of life on mental health, particularly in MHD patients who often contend with elevated levels of depression and anxiety due to the influence of the disease and its treatment (46, 47). The long-term treatment regimen places greater economic and life pressure on MHD patients, may negatively influence their flourishing (48). Regression analysis underscores that quality of life has the most substantial impact on flourishing, highlighting the critical need for interventions aimed at alleviating the burden of disease and improving quality of life.

#### 4.2.3 Psychological factors

Personality traits were found to significantly influence flourishing, with extroversion, conscientiousness, and openness being positively associated with higher flourishing levels. Neuroticism scores were reverse-coded (i.e., higher scores reflect emotional stability), resolving the apparent contradiction. Neuroticism was negatively associated with flourishing. Hypothesis 3 were proved. Personality traits are known to shape daily behaviors and mental health outcomes, with extroverted and conscientious individuals more likely to experience strong mental health (49). The univariate analysis of this study revealed that extroversion among the Big Five personality traits of maintenance hemodialysis patients had a higher correlation with the dimension of positive experience, while conscientiousness and openness had a higher correlation with the dimension of self-accomplishment. Extroversion is characterized by sociability, talkativeness, self-confidence, and preferred emotional expression (50). People receive MHD who are extroverted may experience more positive emotions through social and emotional expression. People with openness are imaginative and creative, eager to try and learn new things, and more likely to adopt new ways of achieving goals (51). Conscientious individuals are organized and prefer to plan, leading to better disease management and goal achievement (52).

Regulatory emotional self-efficacy, the cognitive process by which individuals regulate mood changes in this study, POS and ANG entered the regression equation for the factors associated with flourishing. Zhenghong et al. also showed that regulatory emotional self-efficacy can positively influence well-being (53). Expressing positive emotions is one of the types of disclosure, and individuals who express positive emotions have certain positive tendencies in both explicit and implicit situations and being good at disclosure also plays an important role in the promotion of mental health (54). Patients who can effectively manage their emotions are better equipped to cope with the psychological challenges posed by chronic illness, leading to higher levels of flourishing (55).

Social support was strongly associated with flourishing in all dimensions in this study, further supporting Hypothesis 3 (H3). This implicates that better and broader social relationships are positive affect the level of flourishing, which is in line with the other studies (56, 57). Social support is a major external factor that affects the psychological state and quality of life of patients. Good social support has been shown to predict better emotional states and increase resilience in coping with traumatic events (58, 59). Patients discussing and sharing their feelings with others can receive helpful advice and assistance, which may lead to increased confidence in coping with the illness and ultimately positive emotions (60, 61). Social support provides patients with necessary resources such as emotional, informational, and financial assistance to encourage patients to rethink the meaning of life (62). Therefore, social support has a facilitating effect on flourishing. In this study, family support was more correlated with patients’ flourishing level compared to friends and others support. Family caregivers had the highest percentage of caregivers in this study. The role of family caregivers, who often provide the primary support for MHD patients, was particularly emphasized. They may be burdened by physical, psychological, financial, and time constraints, potentially limiting the support they can provide. (63). Peer support, such as sharing experiences with fellow patients, can offer significant benefits, including information, emotional support, and a sense of community (64), which may enhances quality of life and flourishing.

### 4.3 Implications for practices

The insights garnered from this study underscore the multifaceted nature of flourishing among MHD patients, highlighting the interplay between sociodemographic, disease-related, and psychological factors. Healthcare professionals can leverage these findings to develop comprehensive interventions aimed at enhancing the well-being of MHD patients.

#### 4.3.1 Educational interventions

Given the positive correlation between knowledge of disease and flourishing, tailored educational programs can be designed to enhance patients’ understanding of their condition. These programs should aim to empower patients with knowledge about disease management, coping strategies, and the importance of adherence to treatment protocols.

#### 4.3.2 Psychological support

The significant impact of personality traits and emotional self-efficacy on flourishing suggests the need for psychological interventions. Cognitive-behavioral therapy (CBT) and resilience training can be integrated into patient care to bolster emotional regulation skills, reduce anxiety and depression, and promote positive affect (65). Griva et al. developed an intervention program known as HED-Start. This program integrates CBT, positive psychology, and self-management strategies to assist new patients in successfully transitioning to the hemodialysis phase (66).

#### 4.3.3 Social support

Facilitating support networks is crucial. Healthcare providers can organize support groups where patients share experiences and coping strategies. Additionally, involving family members in educational sessions can strengthen the support system, ensuring that patients receive comprehensive care both within and outside clinical settings (67).

#### 4.3.4 Holistic care

Adopting a patient-centered care approach that integrates physical, psychological, and social aspects of health can lead to better outcomes. Multidisciplinary teams, including nephrologists, psychologists, social workers, and nutritionists, can collaboratively develop and implement individualized care plans.

## 5 Conclusion

The study elucidates the complex interrelations among various factors influencing the flourishing of MHD patients. By identifying key predictors such as quality of life, personality traits, regulatory emotional self-efficacy, and social support, healthcare professionals can devise targeted interventions. Emphasizing a holistic approach that addresses educational, psychological, social, and economic dimensions holds promise in enhancing the well-being and quality of life for patients undergoing maintenance hemodialysis.

## 6 Limitation

While this study offers valuable insights into flourishing among maintenance hemodialysis (MHD) patients, several limitations warrant attention. First, the cross-sectional design inherently precludes causal inferences, necessitating future longitudinal investigations to elucidate the temporal dynamics of flourishing and its predictors among MHD patients. Second, the reliance on non-random sampling methods may constrain the generalizability of the findings, suggesting a need for broader, randomized recruitment strategies in subsequent research. Third, the analytical framework did not incorporate dialysis adequacy indicators (e.g., kt/V), which limits the ability to explore associations between biochemical parameters of dialysis quality and clinical or psychosocial outcomes. To address these gaps, future studies should integrate longitudinal data with biochemical markers such as kt/V to refine predictive models and deepen mechanistic understanding. Additionally, complementing subjective measures with objective assessments (e.g., biomarkers, clinician-rated scales) and contextualizing findings within cultural frameworks could enhance the ecological validity and nuance of interpretations. Collectively, these advancements would provide a more holistic perspective on flourishing in MHD patients, bridging gaps between biomedical parameters, psychosocial constructs, and culturally situated care practices.

## Data Availability

The raw data supporting the conclusions of this article will be made available by the authors, without undue reservation.
