# Supplementary material for: Flourishing and its influencing factors among maintenance hemodialysis patients in Shanghai, China: a cross-sectional study
Source: Front Psychiatry. 2025 Mar 27;16:1480308. doi: 10.3389/fpsyt.2025.1480308 (PMC12000829; doi:10.3389/fpsyt.2025.1480308)
Supplement: Supplementary file 1 [file Table1.docx]

Supplementary Table 1 Value Labels of Characteristics

| Independent Variable | Value Labels |
| --- | --- |
| Education | Elementary school degree or lower=1；Middle school graduate=2；high school graduate=3；College degree or higher=4 |
| Marital status | Married（M_1_=1，M_2_=0，M_3_=0）；Unmarried（M_1_=0，M_2_=1，M_3_=0）；Widowed（M_1_=0，M_2_=0，M_3_=0）；Divorced（M_1_=0，M_2_=0，M_3_=1） |
| Employment | Employed full-time（W_1_=1，W_2_=0，W_3_=0）；Employed part-time（W_1_=0，W_2_=0，W_3_=0）；Retirement（W_1_=0，W_2_=1，W_3_=0）；Unemployment（W_1_=0，W_2_=0，W_3_=1） |
| Number of co-morbid disease | None=0；1=1；2=2；3=3；≥4=4 |
| Degree of knowledge of disease-related information | Very much=4；Much=3；Somehow=2；Not at all=1 |
| Burden of medical expenses for disease | Very light = 1; Mild = 2; Moderate = 3; Severe = 4; Very severe = 5 |
| Impact of disease on life | Very light = 1; Mild = 2; Moderate = 3; Severe = 4; Very severe = 5 |
